# Concomitant Presentation of Acute Acalculous Cholecystitis and Acute Colitis in a Patient with Behcet's Disease

**DOI:** 10.7759/cureus.31295

**Published:** 2022-11-09

**Authors:** Hasan Bakirli, Gultakin Bakirova, Nasser Alhwaymel, Motasem Jaber, Martin Jezovit, Dhilal Issaoui, Martin Nemcek, Ilkin Bakirli, Mohamed Sami, Ifrat Bakirov

**Affiliations:** 1 General and Oncology Surgery Department, Saints Cyril and Methodius University Hospital, Comenius University, Bratislava, SVK; 2 Critical Care Department, King Saud Medical City, Riyadh, SAU; 3 Emergency Department, Al Iman General Hospital, Riyadh, SAU; 4 General Surgery Department, Imam Abdulrahman Alfaisal Hospital, Riyadh, SAU; 5 Gastroenterology Department, Imam Abdulrahman Alfaisal Hospital, Riyadh, SAU; 6 Vascular Surgery Department, National Institute of Cardiovascular Diseases, Slovak Medical University, Bratislava, SVK; 7 Radiology Department, Imam Abdulrahman Alfaisal Hospital, Riyadh, SAU

**Keywords:** aphthous ulcer, us of the abdomen, contrast-enhanced ct of the abdomen and pelvis, inflammatory bowel disease, pericystic fluid, acalculous cholecystitis, colonoscopy, vasculitis, colitis, behcet's disease

## Abstract

In this study, we present a case of Behcet's colitis that caused acute inflammation in the gallbladder and mimicked the clinical picture of an acute abdomen: severe right-sided abdominal pain, nausea, fever, and tenderness in the right hypochondrium, right flank, right loin, and right iliac fossa (RIF), with severely elevated white blood cell (WBC) count. The picture of acute acalculous cholecystitis and acute abdomen was resolved after three days of antibiotic therapy. Then, the pain mainly was localized in the right flank and loin, with mild pain in the right iliac fossa, with positive Rovsing's and psoas signs. The pain in the right flank, loin, and RIF dramatically subsided after initiating a low dose of steroid injections. The colonoscopy, which was performed after the marked improvement of the patient's general condition, showed large, deep ulcers with severe colitis in the proximal transverse colon and the ascending colon. There was no cobblestone appearance. The histopathology of the colonoscopic biopsy showed surface ulceration with marked inflammatory infiltrates, mainly neutrophils, and no granulomas were found. The acid-fast bacillus (AFB) test was reported negative. Detailed history-taking, repeated clinical examinations, laboratory studies, and careful interpretation of ultrasound (US) and contrast-enhanced computed tomography (CECT) findings may prevent unnecessary surgical interventions in such fragile patients and lead to a better prognosis. A diagnosis of Behcet's colitis was made, taking into consideration the patient's past medical history, mucocutaneous lesions, and US, CECT, colonoscopic, and histopathology findings. Although there are no specific investigations and tests for Behcet's colitis, sparing of the rectosigmoid area, the absence of cobblestone appearance, the presence of deep, large round ulcers, patchy localization of the lesions, the absence of granulomas, and negative AFB are helpful for confidently excluding other specific colitis such as Crohn's disease, ulcerative colitis, intestinal tuberculosis (TB), diverticulitis, and ischemic colitis. In our view, in the differential diagnosis of the non-surgical cause of acute abdomen, Behcet's colitis must be considered among other rare causes, such as inferior myocardial infarction, diabetic ketoacidosis, sickle cell disease, familial Mediterranean fever, and acute intermittent porphyria, especially for the population of Mediterranean coast and Middle East countries.

## Introduction

Behcet's disease (BD) is a rare relapsing systemic inflammatory disorder of unknown etiology characterized by recurrent oral ulcers, genital sores, and ocular lesions; however, many other organs, including the vascular, neurological, and musculoskeletal systems, as well as the gastrointestinal (GI) system, can be involved [1-3]. BD was first described by Turkish dermatology professor Hulusi Behcet in 1937 as "recurrent oral aphthous ulcers, genital ulcers, and "hypopyon-uveitis" [4]. The intestinal involvement in BD is rare but important because it is one of the most common causes of fatality and severe morbidity during the disease course [5,6]. For this reason, early diagnosis and appropriate management of gastrointestinal (GI) manifestations of BD are vital to avoid serious complications such as hematochezia, perforation, peritonitis, septicemia, and death. Contrast-enhanced computed tomography (CECT) is the best tool to diagnose or rule out the acute abdomen and gives hints for urgent exploration. In our patient, despite clinical signs of acute abdomen and acute appendicitis, CECT confidently excluded the perforation of the hollow viscus and the inflammation of the appendix. The patient's past medical history, the presence of mucocutaneous lesions in typical locations, and colonoscopic and histopathology findings may help successfully diagnose BD and exclude other inflammatory conditions of the GI tract, such as Crohn's disease, ulcerative colitis, intestinal tuberculosis (TB), diverticulitis, and ischemic colitis. However, there are no specific investigations and tests yet for BD. The current management of the intestinal form of BD is similar to the management of inflammatory bowel diseases and includes aminosalicylates and corticosteroids. For the severe form of Behcet's colitis, corticosteroids are the best choice during acute episodes. During therapy, the patient should be monitored continuously to avoid surgery delays if there are indications of uncontrolled lower GI bleeding, intestinal obstruction, perforation, peritonitis, and failure of conservative treatment. Delayed surgery may result in the poorest outcome. Recently, immunomodulatory agents (thalidomide and azathioprine) and monoclonal antibodies (infliximab and adalimumab) were also successfully introduced to the treatment regimen of intractable forms of BD [7].

## Case presentation

**Figure 1:**
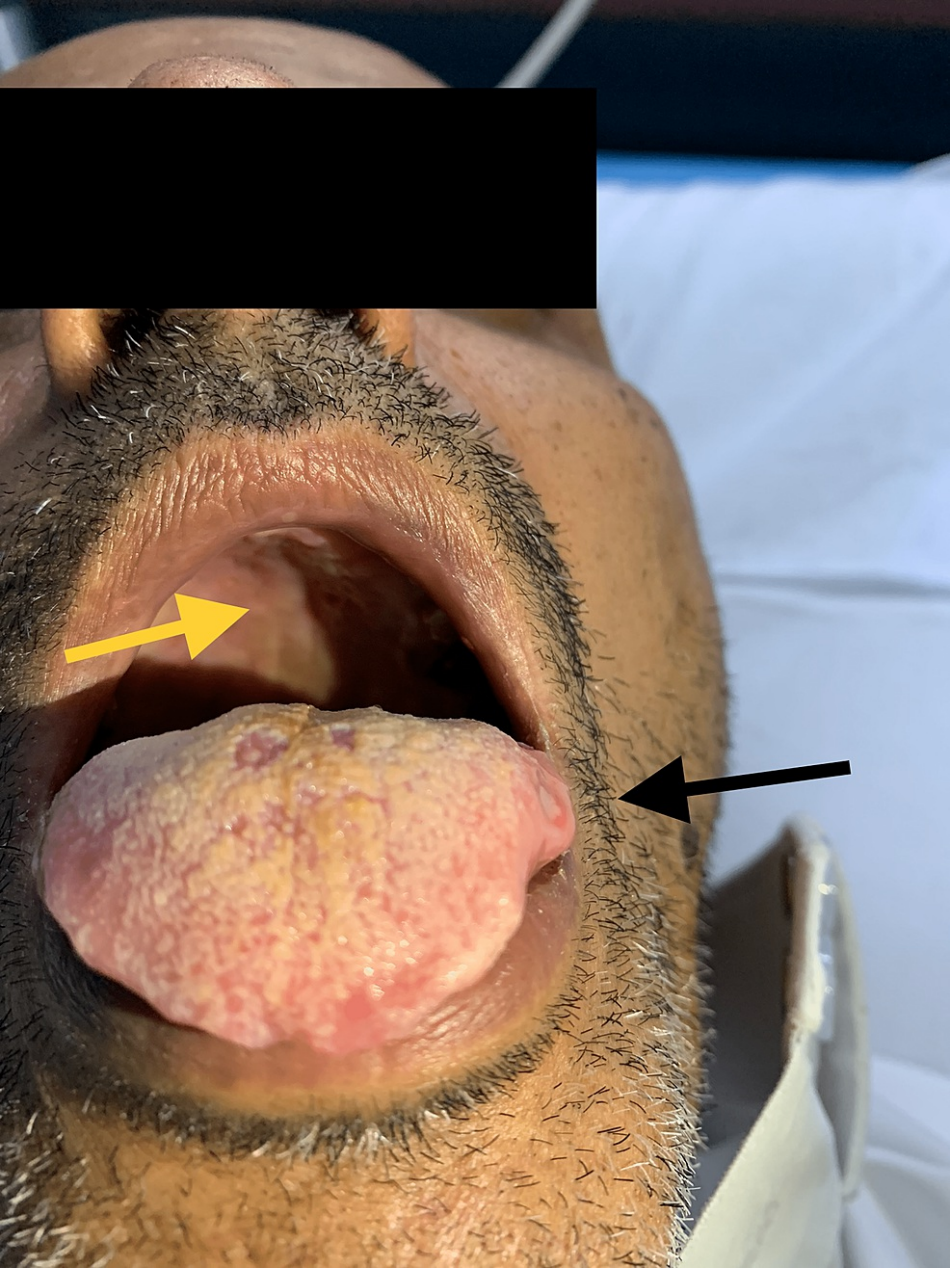
Hard palate lesions (yellow arrow) and ulcer on the left lateral tongue (black arrow).

**Figure 2:**
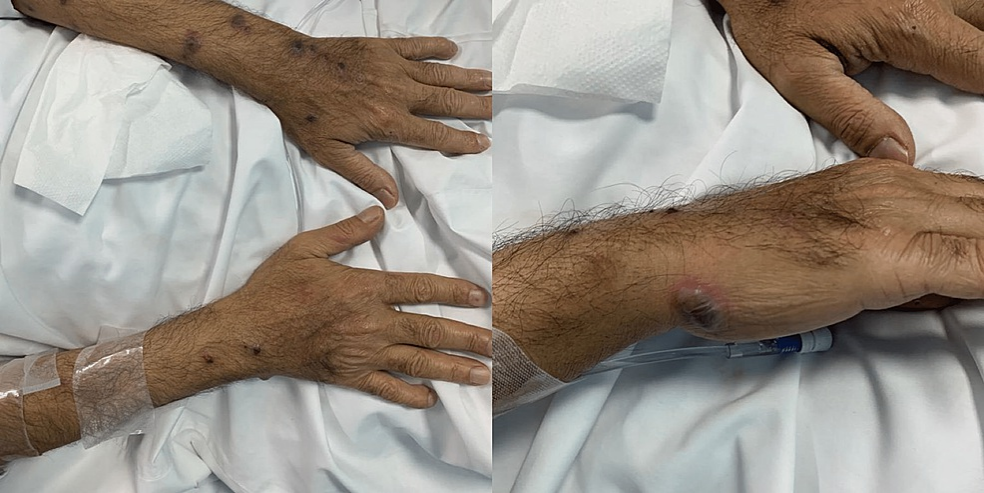
Cutaneous manifestations of Behcet's disease in the hands and wrists.

We describe a case of Behcet's disease that manifested with simultaneous presentation of acute acalculous cholecystitis and acute colitis and mimicked the clinical picture of acute abdomen. The patient is a 51-year-old male who came to the emergency department complaining of severe pain in the right side of the abdomen. He is a known case of epilepsy on phenytoin tablets and Behcet's disease on colchicine tablets. He was diagnosed with epilepsy during childhood that was well controlled by phenytoin tablet 100 mg eight hourly. Fifteen years ago, he was investigated in a tertiary care center for recurrent painful mouth lesions and limbs and genital ulcers, diagnosed with Behcet's disease, and prescribed colchicine and prednisolone tablets. The patient had recurrent hospital visits for mucocutaneous ulcers, and he stopped the prednisolone tablet and continued with colchicine 0.5 mg twice daily. On arrival at the emergency department, the patient had severe right upper abdominal pain and fever. He had nausea as well, but no vomiting and diarrhea. Generally, the patient was looking severely ill, dehydrated, and in intense pain. Clinical examinations revealed ulcers in the mouth, especially in the hard palate (Figure 1), in both hands and wrists (Figure 2), and over the scrotum.

Abdominal examination revealed severe tenderness over the right hypochondrium with mild to moderate guarding and positive Murphy's sign. Furthermore, he has moderate tenderness over the right flank, right loin, and right iliac fossa (RIF), with slight positive rebound tenderness. Appendicular signs (Rovsing's and psoas signs) were positive as well.

The patient received analgesics and underwent a blood workup and an abdominal ultrasound (US). The white blood cell (WBC) count was 25,000/μL (normal value (NV): 3,000-11,000/μL), neutrophils were 22,000/μL (NV: 1,800-7,700/μL), monocytes were 2,300/μL (NV: 200-900/μL), hemoglobin (Hgb) was 11.3 g/dL (NV: 13.1-17.3 g/dL), and red blood cell count was 3.97 × 10^12^/L (NV: 4.2-5.6 × 10^12^/L). Kidney function tests, serum electrolytes (Na, K, and Cl), liver function tests (alanine aminotransferase (ALT), aspartate aminotransferase (AST), direct and total bilirubin, and alkaline phosphatase (ALP)), and serum amylase levels were normal; the serum albumin level was slightly decreased at 28 g/L (NV: 34-50 g/L). Venous blood gas (VBG) showed mild metabolic acidosis at pH 7.32, partial pressure of carbon dioxide (PCO2) of 34 mmHg, bicarbonate of 18 mmol/L, and base deficit of -7. The erythrocyte sedimentation rate (ESR) was significantly raised by 150 mm/hour (NV: 3-15 mm/hour). C-reactive protein (CRP) was positive. Chest X-ray in an erect position did not reveal any chest pathology or free air under the diaphragm. On abdominal X-ray, one loop of jejunum was mildly dilated and appeared as a sentinel loop.

**Figure 3:**
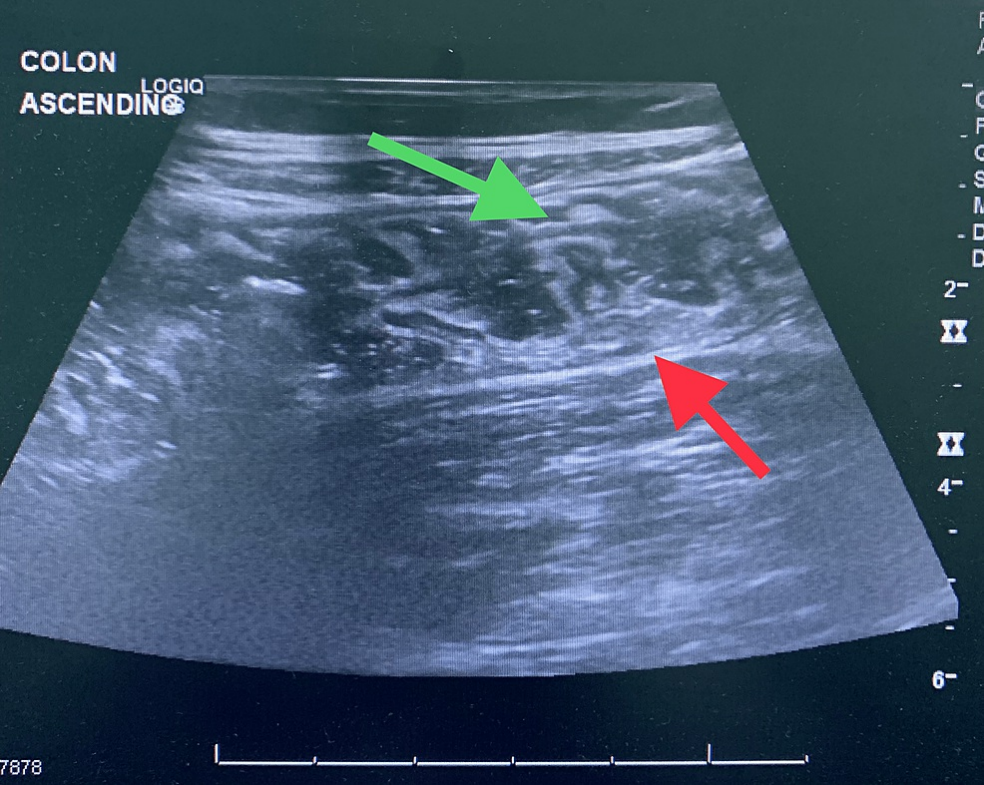
Ultrasound image of Behcet's colitis: thick-walled (red arrow) ascending colon with edematous mucosa (green arrow).

Ultrasound (US) of the abdomen reported an increased thickness of the gallbladder wall up to 9 mm (NV: 2-3 mm), with minimal free fluids in pericystic, subhepatic areas and right iliac fossa. No gallstones were detected, and the common bile duct was within the normal range, at 4 mm only. There was an increased bowel thickening up to 7 mm on the right side of the abdomen in approximately 10 cm segment (Figure 3), and the appendix could not be visualized.

**Figure 4:**
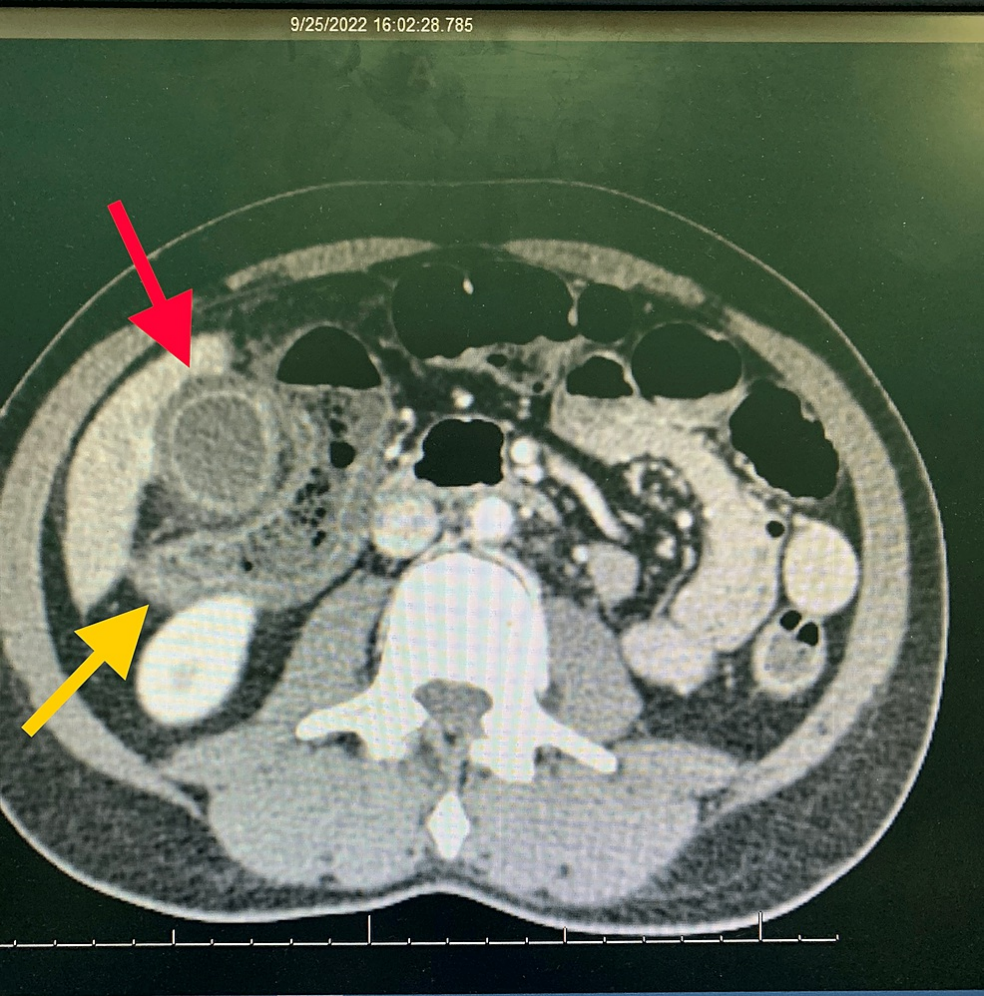
Contrast-enhanced computed tomography showing a severely inflamed gallbladder (red arrow) and inflamed hepatic flexure of the colon (yellow arrow).

Contrast-enhanced computed tomography reported edematous thickening of the gallbladder wall with fat stranding and free fluid in the fatty tissue surrounding the gallbladder and nearby part of the ascending colon and hepatic flexure (Figure 4).

No gallstones were detected. The ascending colon and hepatic flexure look thick-walled due to edema. A minimal amount of free fluid was detected in the pelvis. The appendix is normal and located in a retrocecal position. Other abdominal organs, the liver, spleen, and pancreas, were normal. Abdominal visceral vessels and their main branches were patent. No free intraperitoneal air was found. The imaged portions of both lower lung fields are found to be normal as well. The patient has been admitted with acute acalculous cholecystitis and initiated treatment, which includes antibiotics in the form of ceftriaxone and metronidazole, good hydration by lactated Ringer's solution, analgesics, and antiemetics; he was advised to continue colchicine and phenytoin tablets as before. The next day, WBC and neutrophil counts were raised to 28,900/μL and 24,900/μL, respectively. Gentamicin injection was added to the treatment regimen, and the patient gradually improved. After two days, there was no more pain in the upper abdomen and no nausea, and his appetite regained. However, eating was painful due to mouth lesions. Using the straw was helpful in this situation to have adequate enteral nutrition. At this time, the dominating symptom was right loin pain. Clinical examination showed marked improvement, and the abdomen was soft and lax, with no tenderness in the right hypochondrium and negative Murphy's sign. Palpation revealed moderate tenderness in the right loin and right flank and RIF. WBC and neutrophil counts were markedly reduced to 13,700/μL and 10,300/μL, respectively. After four days of admission, US of the abdomen was requested to monitor resolving inflammation in the gallbladder and possibly to find the cause of residual pain in the right loin, flank, and RIF. The US reported that the marked inflammation in the wall of the gallbladder has resolved, with no more wall edema, no gallstones, and no pericholecystic fluid collection found. However, the previously detected increased thickness of the bowel wall on the right side of the abdomen was still seen, and the whole ascending colon was associated with thickened surrounding fat planes, suggestive of colitis; the appendix could not be seen. Therefore, it was assumed that the signs of acute cholecystitis resolved, as per clinical, laboratory, and ultrasound findings. The remaining residual right loin pain is mostly related to Behcet's colitis. After repeated discussions with the patient, he agreed to undergo steroid therapy in the form of hydrocortisone 50 mg intravenously twice daily. At the same time, the patient was prepared for a colonoscopy. The patient's general condition improved further, and his abdominal pain became tolerable and controllable with paracetamol tablets. No more narcotic analgesics and nonsteroidal anti-inflammatory drug (NSAID) injections were required, and his WBC count further decreased and became 7,200/μL.

**Figure 5:**
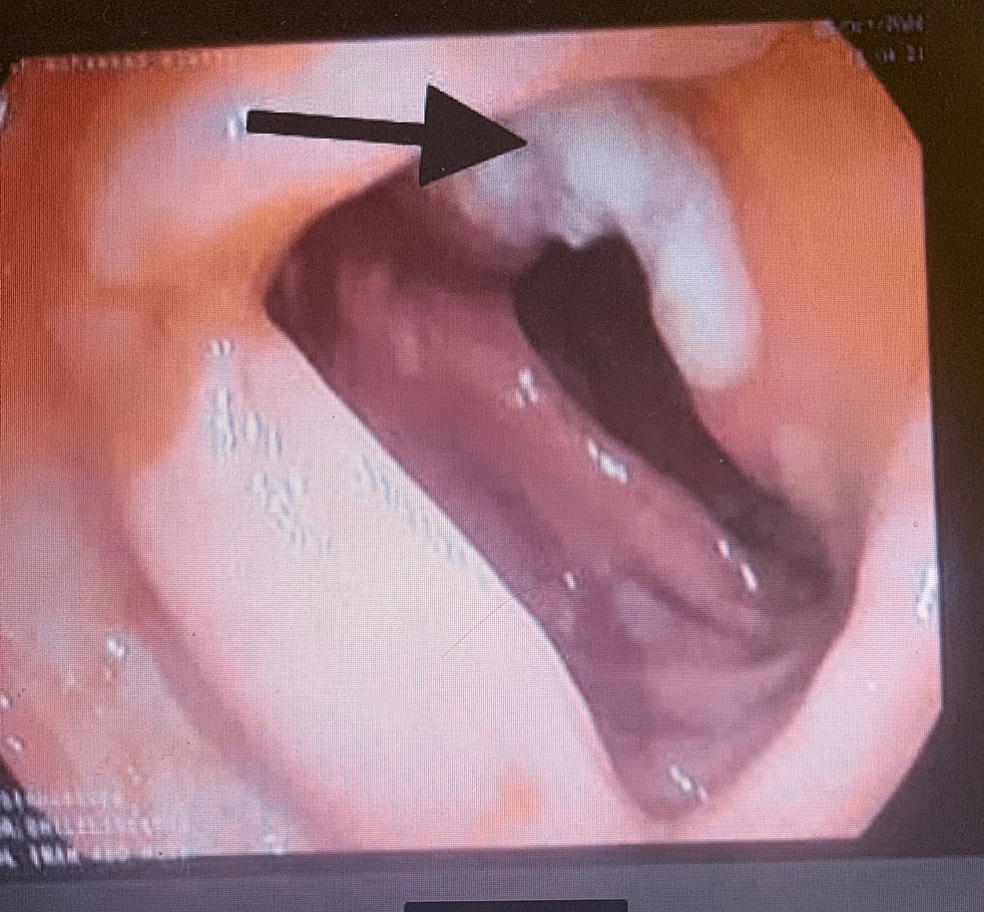
Behcet's colitis with an ulcer at the proximal transverse colon (black arrow).

**Figure 6:**
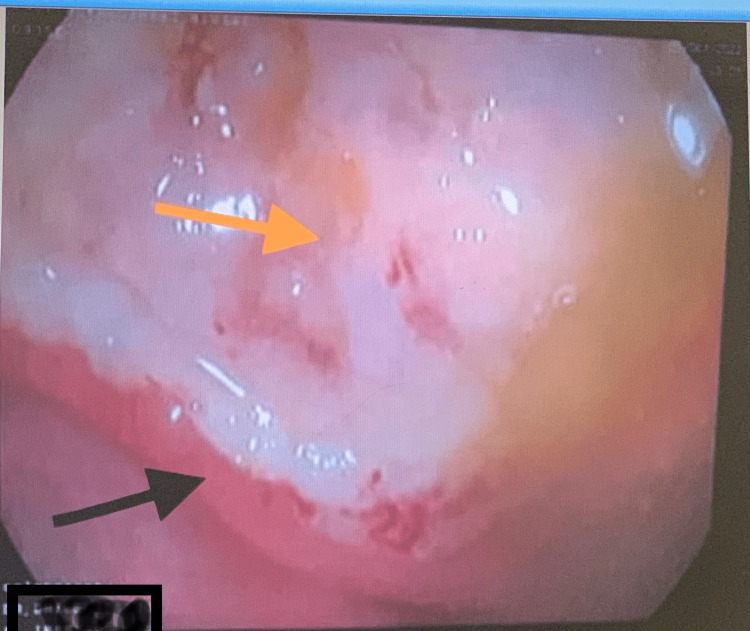
Deep and large ulcer at the ascending colon (yellow arrow) with sharp margins (black arrow).

The colonoscopy was done after 11 days of admission, and the picture of colitis in the descending colon and in the transverse colon was found, with multiple small aphthous ulcers (Figure 5) and severe colitis with deep and large multiple ulcers found in the proximal transverse colon and in the distal part of the ascending colon (Figure 6). Due to perforation risk, no biopsy was taken, and the procedure was aborted.

**Figure 7:**
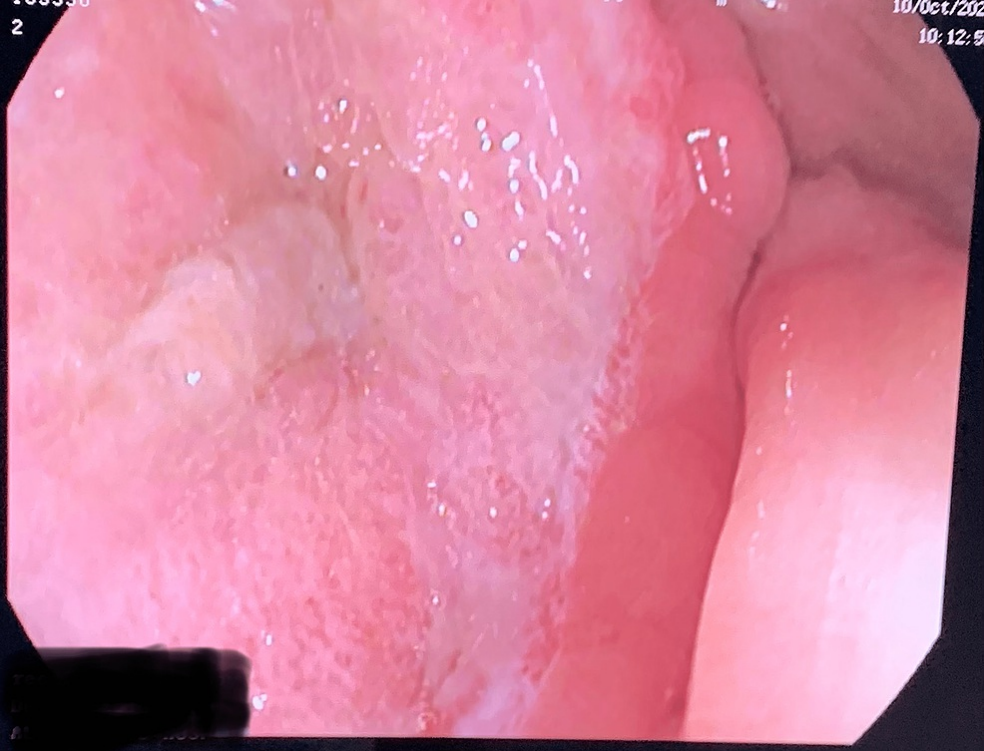
Large and deep ulcer of the ascending colon after a low dose of steroid therapy.

Blood was taken for human immunodeficiency virus (HIV), cytomegalovirus (CMV), and antinuclear antibodies to rule out other possible causes of colonic ulcerations. After five days, just before discharge, the colonoscopy was repeated, and there was a significant improvement in the colonic ulcers (Figure 7).

A biopsy was taken at this moment. Human immunodeficiency virus, cytomegalovirus, lupus factor, and antinuclear antibody tests were negative. The patient has improved and was discharged home in good condition with a low dose of oral steroids and advised to come back for a follow-up of his clinical condition and biopsy result. Transverse colon biopsy showed mild edematous mucosa with a mild mixed inflammatory infiltrate with regularly oriented mucus glands. The section from ascending colonoscopic biopsies revealed surface ulceration comprising vascularized granulation tissue with massive inflammatory infiltrate, mainly neutrophils. The acid-fast bacillus (AFB) stain was negative. The biopsy result was summarized as severe active colitis of the ascending colon, with no granulomas. Considering the patient's history, clinical manifestation, sparing of rectosigmoid involvement, and the absence of cobbling and granulomas on colonoscopy and in pathology, the negative AFB helped rule out Crohn's disease, ulcerative colitis, intestinal TB, diverticulitis, and ischemic colitis. At the two-week follow-up visit, the patient was symptom-free, with a good appetite. The mouth ulcers and hand and scrotal lesions are healed completely; abdominal examination did not reveal any tenderness. The patient was informed about his biopsy result that was sent to his primary hospital for further follow-up.

## Discussion

Acute abdomen can be mimicked by several non-surgical conditions, such as acute inferior myocardial infarction, diabetic ketoacidosis, acute intermittent porphyria, familial Mediterranean fever, and sickle cell disease. Before deciding on laparotomy or laparoscopy, non-surgical causes of the acute abdomen must be eliminated. Our case shows that even acute concomitant manifestations of cholecystitis and colitis in Behcet's disease can mimic the picture of an acute abdomen. Behcet's disease may manifest with several gastrointestinal presentations. They are summarized by Wu et al. (2012) in their study as per literature information (Table 1) [7].

**Table 1:** Gastrointestinal manifestations of Behcet's disease.

Reference	Type of manifestation	Symptom	Frequency	Complication	Treatment	Outcome
Zhang et al. [8]	Recurrent oral ulcer	Painful ulcer	Almost 100%	Rare	Topical measures	Excellent
Tursen et al. [9]						
Mori et al. [10]	Esophageal ulcer/esophagitis	Substernal pain, dysphagia	Very rare	Very rare	Corticosteroid	Excellent
Yi et al. [11]						
Ning-Sheng et al. [12]	Gastric ulcer and/or duodenal ulcer	Epigastric pain	Variable (very rare to 45%)	Very rare	Uncertain	Excellent
Choi et al. [13]	Small and/or large intestinal ulcers	Abdominal pain, hematochezia	Variable (1.4%-16%)	Rare (perforation, massive bleeding)	Sulfasalazine, corticosteroid, azathioprine, tumor necrosis factor-α antagonist, thalidomide	Good
Köklü et al. [14]						
Bayraktar et al. [15]	Large artery aneurysm/thrombosis in the abdomen	Infarction, ischemia	Very rare	Very rare	Corticosteroid azathioprine, cyclophosphamide	Poor
Chubachi et al. [16]						
Bismuth et al. [17]	Large vein thrombosis in the abdomen	Budd-Chiari syndrome	Very rare	Very rare	Corticosteroid, azathioprine	Poor

A detailed past medical history, repeated clinical examinations, interpretation of US and CECT findings in correlation with clinical signs, and consideration of the response for appropriate conservative management might help the surgeon avoid unnecessary surgical intervention in such a fragile group of patients, who may develop serious complications related to anesthesia and surgical trauma and stress. In our patient, acute non-calculous cholecystitis most likely was due to an inflammatory reaction secondary to acute inflammation of the nearby colon. CT images showed that the inflammatory plane between the right side colon and the gallbladder was continuous. Also, clinical and ultrasound signs of cholecystitis were resolved rapidly, denoting the possible reactionary origin of cholecystitis rather than a direct complication of BD. However, both sequences are possible due to the patient's poor immunity. In the current literature, we have not found acute cholecystitis among abdominal manifestations of BD, which also indirectly may support our idea that acute cholecystitis probably appeared as a reaction to nearby severe colonic inflammation. Behcet's colitis is treated with sulfasalazine, corticosteroid, azathioprine, TNF-α antagonist, and thalidomide. Our patient remained in pain despite triple antibiotics and rapidly improved after initiating a low dose of steroid injection in the form of hydrocortisone 50 mg intravenously twice daily.

## Conclusions

Behcet's colitis can cause inflammation in the gallbladder and may mimic the picture of an acute abdomen. In the differential diagnosis of acute abdomen, Behcet's colitis should be considered among other uncommon non-surgical causes of acute abdomen such as myocardial infarction, diabetic ketoacidosis, sickle cell disease, familial Mediterranean fever, and acute intermittent porphyria, especially for the population of the Mediterranean coast and the Middle East. To the best of our knowledge, this is the first published case of acute acalculous cholecystitis concomitantly manifesting with severe acute colitis in a patient with Behcet's disease.
